# Evaluation of a Novel MALDI Biotyper Algorithm to Distinguish *Mycobacterium intracellulare* From *Mycobacterium chimaera*

**DOI:** 10.3389/fmicb.2018.03140

**Published:** 2018-12-18

**Authors:** L. Elaine Epperson, Markus Timke, Nabeeh A. Hasan, Paul Godo, David Durbin, Niels K. Helstrom, Gongyi Shi, Markus Kostrzewa, Michael Strong, Max Salfinger

**Affiliations:** ^1^Center for Genes, Environment and Health, National Jewish Health, Denver, CO, United States; ^2^Bruker Daltonik GmbH, Bremen, Germany; ^3^Mycobacteriology Laboratory, National Jewish Health, Denver, CO, United States; ^4^Bruker Daltonics, Billerica, MA, United States; ^5^Department of Medicine, National Jewish Health, Denver, CO, United States; ^6^College of Public Health, University of South Florida, Tampa, FL, United States

**Keywords:** nontuberculous mycobacteria, diagnostics, MALDI, *Mycobacterium chimaera*, *Mycobacterium intracellulare*, species identification, MAC, NTM

## Abstract

Accurate and timely mycobacterial species identification is imperative for successful diagnosis, treatment, and management of disease caused by nontuberculous mycobacteria (NTM). The current most widely utilized method for NTM species identification is Sanger sequencing of one or more genomic loci, followed by BLAST sequence analysis. MALDI-TOF MS offers a less expensive and increasingly accurate alternative to sequencing, but the commercially available assays used in clinical mycobacteriology cannot differentiate between *Mycobacterium intracellulare* and *Mycobacterium chimaera*, two closely related potentially pathogenic species of NTM that are members of the *Mycobacterium avium* complex (MAC). Because this differentiation of MAC species is challenging in a diagnostic setting, Bruker has developed an improved spectral interpretation algorithm to differentiate *M. chimaera* and *M. intracellulare* based on differential spectral peak signatures. Here, we utilize a set of 185 MAC isolates that have been characterized using *rpoB* locus sequencing followed by whole genome sequencing in some cases, to test the accuracy of the Bruker subtyper software to identify *M. chimaera* (*n* = 49) and *M. intracellulare* (*n* = 55). 100% of the *M. intracellulare* and 82% of the *M. chimaera* isolates were accurately identified using the MALDI Biotyper algorithm. This subtyper module is available with the MALDI Biotyper Compass software and offers a promising mechanism for rapid and inexpensive species determination for *M. chimaera* and *M. intracellulare*.

## Introduction

The use of Matrix Assisted Laser Desorption Ionization-Time of Flight Mass Spectrometry (MALDI-TOF MS) for identification of microbial specimens has scaled up dramatically over the past decade due to the continual improvement of available tools, including expanded spectral peak databases [reviewed in Doern and Butler-Wu (2016); Rahi et al. (2016); and Alcaide et al. (2018)]. Species identification of bacteria is now honed to strain-specific and subspecies distinctions in many cases (Neville et al., 2011; Huang et al., 2018) as well as the characterization of non-bacterial organisms such as fungi (Chalupova et al., 2014; Levesque et al., 2015). These approaches are relatively rapid, inexpensive, and accurate for use in clinical diagnostics (Neville et al., 2011), environmental sample analysis, food safety, and other applications (Carbonnelle et al., 2011; Florio et al., 2018). MALDI-TOF MS is increasingly utilized to distinguish clinically relevant nontuberculous mycobacteria (NTM) (Levesque et al., 2015; Costa-Alcalde et al., 2018).

The *Mycobacterium avium* complex (MAC) comprises several clinically important mycobacterial species including *M. avium* (subsp. *hominissuis*), *M. intracellulare*, and *M. chimaera* (Diel et al., 2018; Forbes et al., 2018), among others [e.g., *M. colombiense* (Murcia et al., 2006) and *M. yongonense* (Castejon et al., 2018)]. Although *M. avium* and *M. intracellulare* species are more frequently observed in clinical cases, a recent series of *M. chimaera* infections originating in cardiac surgery suites (van Ingen et al., 2017) illuminates the importance of identifying members of the MAC complex at the species level in a rapid and accurate manner. While the spectrum of *M. avium* is relatively distinct from the other two species, *M. intracellulare* and *M. chimaera* are highly similar to each other (van Ingen et al., 2012), and not readily distinguished using the standard approaches of most MALDI-TOF peak interpretation algorithms (Boyle et al., 2015a; Leyer et al., 2017). Pulmonary infections caused by the different MAC species are distinct in their virulence and clinical features, indicating that species-level identification is important for effective prognostics (Boyle et al., 2015b; Kim et al., 2017). Bruker Daltonik (Bremen, Germany) developed a commercially available algorithm to differentiate *M. chimaera* and *M. intracellulare* from each other using only MALDI-TOF peak data. This algorithm was found to perform well against 59 bacterial isolates of European origin using the internal transcribed spacer (ITS) sequence to identify species (Pranada et al., 2017). Here we set out to evaluate the same MALDI algorithm against 111 isolates of United States origin. Sequence from the *rpoB* locus was used to determine species, but in 16 discrepant cases, the isolates were whole genome sequenced and species identity was determined using phylogenomics.

## Materials and Methods

### NTM Species Determination Using Sanger Sequencing

All samples were of clinical origin within the United States, either bronchoalveolar lavage, sputum, or tissue. Isolates were sent to National Jewish Health for NTM species identification and/or antimicrobial susceptibility testing. DNA from these isolated cultures of 300 acid-fast bacilli were analyzed using a targeted 711 base pair region of the *rpoB* locus that was Sanger sequenced using ABI 3730xL and queried against the National Center for Biotechnology Information (NCBI) Basic Local Alignment Search Tool (BLAST) database. The *rpoB* target is one of several recommended for NTM species determination according to current national clinical microbiology guidelines (Forbes et al., 2018); other targets that are utilized for this purpose are 16S rRNA, secA, ITS, and hsp65 (Lecorche et al., 2018). From this collection, a subset (185) were identified as one of the MAC species according to rpoB sequence, i.e., *M. avium* (*n* = 74*), M. intracellulare* (*n* = 55), or *M. chimaera* (*n* = 56).

### MALDI-TOF MS

MAC strains were subcultured onto Middlebrook 7H11 agar media and incubated for 7–10 days to obtain sufficient biomass for MALDI-TOF MS identification. Colonies from the 7H11 plates were harvested and heat-killed in high performance liquid chromatography (HPLC) grade water, followed by acetonitrile/formic acid extraction procedure according to the manufacturer protocol. The MALDI-TOF target was spotted with 1 μL of extract supernatant and overlaid with 1 μL of α-Cyano-4-hydroxycinnamic acid (HCCA) matrix prior to data capture using the Bruker MALDI-TOF Biotyper system (software v4.0). A minimum score of 1.8 was required. A complete MALDI spectrum dataset was sent to Germany for MALDI spectrum analysis in parallel at Bruker Daltonik in Bremen, Germany.

For samples identified by MALDI-TOF MS as *M. chimaera-intracellulare* group, a second-tier analysis was performed using a recently developed research subtyping software from Bruker Daltonik [(Pranada et al., 2017) herein referred to as MBT Subtyping Module]. One sample, NTM-184, was eliminated from the sensitivity and specificity calculations because the MALDI results placed it in the *M. chimaera-intracellulare* group, but the *rpoB* result was *M. avium*, and this sample did not have whole genome sequence (WGS) information (see below.) An additional seven samples, six of which were identified as *M. chimaera* using *rpoB*, were also eliminated from these calculations because the MBT Subtyping Module did not yield a result. These strains are listed in Table 1 under the MBT Subtyper as “*M. chim/M. int.*” It should be noted that the MBT Subtyping Module returned a high score on these seven samples but because it was unable to distinguish the two species, it does not make a call in these cases.

**Table 1:** Comparison of single locus sequencing results to whole genome sequencing results for sixteen MAC strains for each sample that was whole genome sequenced, three loci were extracted and analyzed by BLAST to the non-redundant NCBI database giving the results listed.

	Region extracted from WGS data					
Sample	16S (1026bp)	16S 403^∗^	ITS (335-336bp)	rpoB (752bp)	MBT subtyper	WGS
NTM-006	*M. chim/M.int*	*M. int*	*M. chim/M.int*	*M. chim*	*M. int*	*M. int*
NTM-168	MAC	*M. int*	*M. chim/M.int*	*M. int*	*M. chim/M.int*	*M. int*
NTM-224	*M. mars/M.int*	*M. chim*	*M. int*	*M. chim*	*M. chim/M.int*	*M. chim*
NTM-105	MAC	*M. int*	*M. chim/M.int*	*M. chim*	*M. int*	*M. chim*
NTM-206	MAC	*M. int*	*M. chim/M.int*	*M. chim*	*M. int*	*M. chim*
NTM-035	MAC	*M. int*	missing data	*M. chim*	*M. int*	*M. chim*
NTM-232	MAC	*M. int*	*M. chim/M.int*	*M. chim*	*M. int*	*M. chim*
NTM-019	*M. chim/M.int*	*M. int*	*M. chim/M.int*	*M. chim*	*M. int*	*M. chim*
NTM-178	MAC	*M. int*	*M. chim/M.int*	*M. chim*	*M. int*	*M. chim*
NTM-107	MAC	*M. int*	*M. chim/M.int*	*M. chim*	*M. int*	*M. chim*
NTM-054	*M. mars/M.int*	*M. chim*	*M. int/M.int yong*	*M. chim*	*M. int*	*M. chim*
NTM-223	MAC	*M. int*	*M. int*	*M. chim*	*M. chim/M.int*	*M. chim*
NTM-203	MAC	*M. int*	*M. int*	*M. chim*	*M. chim/M.int*	*M. chim*
NTM-208	MAC	*M. int*	*M. int*	*M. chim*	*M. chim/M.int*	*M. chim*
NTM-204	MAC	*M. int*	*M. int*	*M. chim*	*M. chim/M.int*	*M. chim*
NTM-230	*M. chim*	*M. chim*	*M. chim*	*M. chim*	*M. chim/M.int*	*M. chim*

*Also listed are the species as determined using only the 403 SNP from 16S, assuming a strain was previously identified as belonging to the chimaera/intracellulare group. MBT Subtyper result and the WGS species call based on phylogenomic tree. M. chim = M. chimaera, M. int = M. intracellulare, M. yong = M. yongonense, M. mars = M. marseillense.*

### Phylogenomic Analysis of Ambiguous NTM Strains

For almost all samples, the *rpoB* species identification was considered to be the actual species of that strain. In a few cases, samples yielded discrepant results, and for these samples, WGS was used to assign a species identification to a given mycobacterial strain. Genomic DNA was isolated according to a protocol adapted from Käser et al. (2010), employing a column DNA clean in lieu of a phenol chloroform extraction and alcohol precipitation. Genomic libraries were constructed using Nextera XT and sequenced using Illumina chemistry (Illumina, Inc., San Diego, CA).

Illumina reads were trimmed of adapters and low quality bases (< Q20) using Skewer (Jiang et al., 2014). Trimmed reads were assembled into scaffolds using Unicycler (Wick et al., 2017), and genome assemblies were compared against a selection of reference genomes to calculate average nucleotide identities (ANI, chjp/ANI on GitHub.com, Supplementary Table S1) and to assign a species call to each isolate (Goris et al., 2007; Richter and Rossello-Mora, 2009). Trimmed reads and background reference genomes were mapped to the *M. chimaera* CDC 2015-22-71 genome (Hasan et al., 2017) using Bowtie2 software (Langmead and Salzberg, 2012). Single nucleotide polymorphisms (SNPs) were called using SAMtools mpileup program (Li et al., 2009), and a multi-fasta sequence alignment was created from concatenated basecalls from all strains (Davidson et al., 2014; Page et al., 2016). Resulting sequences were used to make a maximum likelihood (ML) phylogenetic tree using RAxML-NG with a GTR+G substitution model (Kozlov et al., 2018) and 1,000 bootstrap replicates. The tree was annotated and visualized with ggtree (Yu et al., 2017). Reference genomes for the tree are, for *M. chimaera*, listing genome name, accession number: AH16, CP012885.2; CDC 2015-22-71,CP019221.1; DSM 44623, CP015278.1; SJ42, CP022223.1; ZUERICH-2, CP015267.1; LJHL01, LJHL00000000.1; LJHM01, LJHM00000000.1; LJHN01, LJHN00000000.1; 1956, CP009499.1; ATCC 13950, NC_016946.1; MOTT-02, NC_016947.1; MOTT-64, NC_016948.1; and for *M. intracellulare*: 1956, CP009499.1; ATCC 13950, NC_016946.1; MOTT-02, NC_016947.1; MOTT-64, NC_016948.1.

The whole genome sequence data are available at NCBI in Bioproject number PRJNA506060^1^ with the following accession numbers: NTM-006, SAMN10441865; NTM-019, SAMN10 441945; NTM-035, SAMN10441946; NTM-054, SAMN1044 1947; NTM-105, SAMN10441948; NTM-107, SAMN10441949; NTM-168, SAMN10441950; NTM-178, SAMN10441951; NTM-203, SAMN10441952; NTM-204, SAMN10441953; NTM-206, SAMN10441954; NTM-208, SAMN10441955; NTM-223, SAMN 10441956; NTM-224, SAMN10441957; NTM-230, SAMN10 441958; NTM-232, SAMN10441959.

### Comparison of WGS to Single Locus Species Determination

Using primer sequences of known targets, three regions were extracted from the WGS data. These were previously targeted regions from the following loci: 16S rDNA (Springer et al., 1996; van Ingen et al., 2012), the 16S–23S internal transcribed spacer (ITS) (Schweickert et al., 2008), and *rpoB* (Adekambi et al., 2003; Ben Salah et al., 2008). These sequences were used to BLAST against the non-redundant NCBI database and the top species call or calls are reported in Table 1.

## Results

From the initial strain collection, 185 isolates were identified by *rpoB* amplicon sequencing as one of the three prominent clinical MAC species, specifically 74 *M. avium*, 55 *M. intracellulare*, and 56 *M. chimaera.* These 185 strains were then analyzed using MALDI-TOF MS at National Jewish Health, in Denver, Colorado, United States and the data were re-analyzed in Bremen, Germany, yielding identical results according to the Biotyper 4.0 software. Of the 74 *M. avium* isolates, 73 were identified as *M. avium*. A single *M. avium* sample (as identified by *rpoB*) and the remaining 111 samples were all identified as being in the *M. chimaera-intracellulare* group.

These 112 strains were then subjected to a second tier of analysis wherein their MALDI-TOF MS spectra were evaluated using the recently developed MBT Subtyping Module software; see Methods (Pranada et al., 2017). One sample, NTM-006, was identified as *M. chimaera* by *rpoB* but as *M. intracellulare* by WGS, thus the sample was categorized as *M. intracellulare*. This was the only sample for which WGS yielded a result that differed from *rpoB*. In this case, the WGS result was considered to be the actual species for calculations. After removing the eight samples that were unconfirmed (see Methods), the samples numbered 55 *M. intracellulare* and 49 *M. chimaera* that were suitable to evaluate the MBT Subtyping Module algorithm for its ability to distinguish these two species.

**FIGURE 1:**
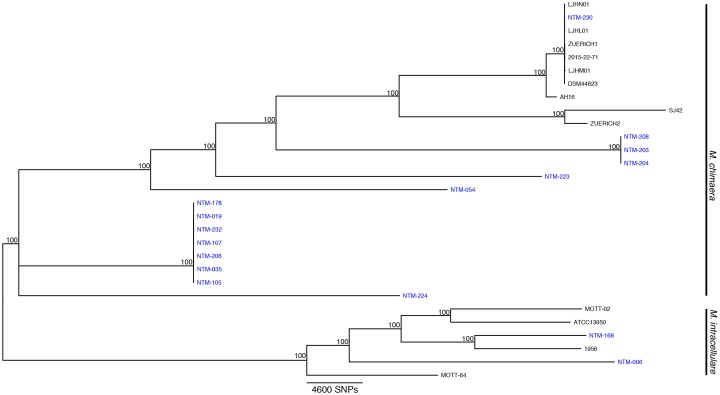
*Mycobacterium chimaera and Mycobacterium intracellulare* whole genome phylogenomic tree. Available whole genome data from *M. chimaera* and *M. intracellulare* were used to visualize the relationships of 16 strains for this study (in blue) and 13 reference strains in comparison to the reference genome of *M. chimaera* CDC 2015-22-71. Bootstrap support values are given at nodes, scale bar for SNP distances is at bottom of figure.

Sixteen isolates were selected for WGS. These strains were investigated in greater depth using extracted sequences of specific loci (Table 1) in addition to phylogenomic tree analysis using the WGS data (Figure 1). The specific loci were selected to reflect the regions that are most commonly used for mycobacterial species identification. For most Mycobacteria spp., these loci are useful for identifying to the NTM species level. But the close relationship of *M. chimaera* and *M. intracellulare* is apparent in these genes since the two species’ sequences over these regions are largely interchangeable, consistent with the initial confounding observations that led to the naming of *M. chimaera* (Tortoli et al., 2004). Based on these extracted sequences, the *rpoB* fragment was most reflective of the WGS result (Table 1).

For the 49 samples identified by sequencing as *M. chimaera*, the MBT Subtyping Module identified 40 as *M. chimaera* and 9 as *M. intracellulare* for a sensitivity of 81.6%. However, because all of the Subtyper *M. chimaera* calls were in fact *M. chimaera* according to whole genome sequencing, the positive predictive value for a call of *M. chimaera* is 100%. The *M. intracellulare* (*n* = 55) data differed in that all 55 samples were identified as *M. intracellulare* for a sensitivity of 100%, but nine of the *M. chimaera* samples were called *M. intracellulare* by the Subtyping software, reducing the specificity, and the positive predictive value is 86% or 55/(55+9). This indicates that a minority (∼18%, or 9/49) of the true *M. chimaera* samples would be recognized as *M. intracellulare* using the MALDI Biotyper Compass software.

## Discussion

The recent outbreak of *M. chimaera*-contaminated heater-cooler devices used in cardiac surgery has increased the clinical urgency for differentiating *M. chimaera* from *M. intracellulare*. The MALDI-TOF MS approach to species identification is less expensive and more rapid than sequencing methods, but until now has been unable to distinguish these two closely related MAC species. Our findings indicate that a call of *M. chimaera* by the Bruker subtyping algorithm tested here is correct every time for a positive predictive value of 100%. We also found that a call of *M. intracellulare* is correct 86% of the time, that is, 14% of our samples that were recognized as *M. intracellulare* by MALDI are actually more closely related to *M. chimaera* than to *M. intracellulare*. Given the regional diversity of NTM species, it is possible that these strains were not available in the initial European sample set during test design. A number of these strains cluster more closely with the *M. chimaera* reference strains (samples 178, 019, 232, etc.), although they are arguably relatively distant from any available reference. The dynamic nature of bacterial phylogenomics requires us to integrate currently available references and whole genome approaches with an understanding that species nomenclature is always evolving as new branches and clades become apparent.

With increasing numbers of *M. chimaera* and *M. intracellulare* isolates requiring species identification in diagnostic reference laboratories, it is clear that improved tools are needed to quickly and accurately identify and distinguish these close mycobacterial species, in most cases inseparable using average nucleotide identity measures (Supplementary Table S1). We observe a qualitative difference in genome size for these two species and performed a statistical comparison of available complete reference genomes from *M. chimaera* (mean ± 1s.d., 6.24 ± 0.29Mb, *n* = 8) and *M. intracellulare* (5.52 ± 0.12Mb, *n* = 6), and found that *M. chimaera* was consistently larger by ∼700,000 bases (*p* < 0.001, Mann-Whitney U test.) This information may provide a path to discovering markers that can distinguish the two species, but the standard and reliable tools used to distinguish most NTM from one another often fail to discriminate between these very closely related strains. The discerning capability of the Bruker Subtyper module with MALDI spectral data offers an available and notable advance in MAC species identification.

## Author Contributions

MT, MK, GS, and MaS designed the study. LEE wrote the manuscript and performed whole genome sequencing. NKH, PG, and DD performed *rpoB* sequencing and MALDI-TOF analysis. MT, MK, and GS performed parallel MALDI-TOF spectral analysis and developed and implemented the subtyping software. NAH analyzed whole genome sequencing data, extracted loci of interest, and created visualizations. All authors contributed to writing, editing, and overall presentation of the manuscript.

## Conflict of Interest Statement

MT and MK are employed by Bruker Daltoniks and GS by Bruker Daltonics. The remaining authors declare that the research was conducted in the absence of any commercial or financial relationships that could be construed as a potential conflict of interest.

## Footnotes

1 https://www.ncbi.nlm.nih.gov/bioproject/PRJNA506060/

## References

[B1] Adekambi T. Colson P. Drancourt M. (2003). rpoB-based identification of nonpigmented and late-pigmenting rapidly growing mycobacteria. *J. Clin. Microbiol.* 41 5699–5708. 10.1128/JCM.41.12.5699-5708.2003 14662964 PMC308974

[B2] Alcaide F. Amlerova J. Bou G. Ceyssens P. J. Coll P. Corcoran D. (2018). Genomics European Study Group on, and Diagnosis Molecular. How to: identify non-tuberculous Mycobacterium species using MALDI-TOF mass spectrometry. *Clin. Microbiol. Infect.* 24 599–603. 10.1016/j.cmi.2017.11.012 29174730

[B3] Ben Salah I. Adekambi T. Raoult D. Drancourt M. (2008). rpoB sequence-based identification of *Mycobacterium avium* complex species. *Microbiology* 154 3715–3723. 10.1099/mic.0.2008/020164-0 19047739

[B4] Boyle D. P. Zembower T. R. Qi C. (2015a). Evaluation of Vitek MS for rapid classification of clinical isolates belonging to *Mycobacterium avium* complex. *Diagn. Microbiol. Infect. Dis.* 81 41–43. 10.1016/j.diagmicrobio.2014.09.026 25445119

[B5] Boyle D. P. Zembower T. R. Reddy S. Qi C. (2015b). Comparison of clinical features, virulence, and relapse among *Mycobacterium avium* complex species. *Am. J. Respir. Crit. Care Med.* 191 1310–1317. 10.1164/rccm.201501-0067OC 25835090

[B6] Carbonnelle E. Mesquita C. Bille E. Day N. Dauphin B. Beretti J. L. (2011). MALDI-TOF mass spectrometry tools for bacterial identification in clinical microbiology laboratory. *Clin. Biochem.* 44 104–109. 10.1016/j.clinbiochem.2010.06.017 20620134

[B7] Castejon M. Menendez M. C. Comas I. Vicente A. Garcia M. J. (2018). Whole-genome sequence analysis of the *Mycobacterium avium* complex and proposal of the transfer of *Mycobacterium yongonense* to *Mycobacterium intracellulare* subsp. *yongonense* subsp. nov. *Int. J. Syst. Evol. Microbiol.* 68 1998–2005. 10.1099/ijsem.0.002767 29683417

[B8] Chalupova J. Raus M. Sedlarova M. Sebela M. (2014). Identification of fungal microorganisms by MALDI-TOF mass spectrometry. *Biotechnol. Adv.* 32 230–241. 10.1016/j.biotechadv.2013.11.002 24211254

[B9] Costa-Alcalde J. J. Barbeito-Castineiras G. Gonzalez-Alba J. M. Aguilera A. Galan J. C. Perez-Del-Molino M. L. (2018). Comparative evaluation of the identification of rapidly growing non-tuberculous mycobacteria by mass spectrometry (MALDI-TOF MS), GenoType Mycobacterium CM/AS assay and partial sequencing of the rpobeta gene with phylogenetic analysis as a reference method. *Enferm. Infect. Microbiol. Clin.* 10.1016/j.eimc.2018.04.012 [Epub ahead of print]. 29871765

[B10] Davidson R. M. Hasan N. A. Reynolds P. R. Totten S. Garcia B. Levin A. (2014). Genome sequencing of *Mycobacterium abscessus* isolates from patients in the United States and comparisons to globally diverse clinical strains. *J. Clin. Microbiol.* 52 3573–3582. 10.1128/JCM.01144-14 25056330 PMC4187745

[B11] Diel R. Nienhaus A. Ringshausen F. C. Richter E. Welte T. Rabe K. F. (2018). Microbiologic outcome of interventions against *Mycobacterium avium* complex pulmonary disease: a systematic review. *Chest* 153 888–921. 10.1016/j.chest.2018.01.024 29410162

[B12] Doern C. D. Butler-Wu S. M. (2016). Emerging and future applications of matrix-assisted laser desorption ionization Time-of-flight (MALDI-TOF) mass spectrometry in the clinical microbiology laboratory: a report of the association for molecular pathology. *J. Mol. Diagn.* 18 789–802. 10.1016/j.jmoldx.2016.07.007 27770851

[B13] Florio W. Tavanti A. Barnini S. Ghelardi E. Lupetti A. (2018). Recent advances and ongoing challenges in the diagnosis of microbial infections by MALDI-TOF mass spectrometry. *Front. Microbiol.* 9:1097. 10.3389/fmicb.2018.01097 29896172 PMC5986882

[B14] Forbes B. A. Hall G. S. Miller M. B. Novak S. M. Rowlinson M. C. Salfinger M. (2018). Practice guidelines for clinical microbiology laboratories: mycobacteria. *Clin. Microbiol. Rev.* 31:e00038-17. 10.1128/CMR.00038-17 29386234 PMC5967691

[B15] Goris J. Konstantinidis K. T. Klappenbach J. A. Coenye T. Vandamme P. Tiedje J. M. (2007). DNA-DNA hybridization values and their relationship to whole-genome sequence similarities. *Int. J. Syst. Evol. Microbiol.* 57 81–91. 10.1099/ijs.0.64483-0 17220447

[B16] Hasan N. A. Lawsin A. Perry K. A. Alyanak E. Toney N. C. Malecha A. (2017). Complete genome sequence of *Mycobacterium chimaera* strain CDC2015-22-71. *Genome Announc.* 5:e00693-17 10.1128/genomeA.00693-17 PMC5543635 28774973

[B17] Huang T. S. Lee C. C. Tu H. Z. Lee S. S. (2018). Rapid identification of mycobacteria from positive MGIT broths of primary cultures by MALDI-TOF mass spectrometry. *PLoS One* 13:e0192291. 10.1371/journal.pone.0192291 29394275 PMC5796708

[B18] Jiang H. Lei R. Ding S. W. Zhu S. (2014). Skewer: a fast and accurate adapter trimmer for next-generation sequencing paired-end reads. *BMC Bioinformatics* 15:182. 10.1186/1471-2105-15-182 24925680 PMC4074385

[B19] Käser M. Ruf M. T. Hauser J. Pluschke G. (2010). Optimized DNA preparation from mycobacteria. *Cold Spring Harb. Protoc.* 2010:pdb.prot5408. 10.1101/pdb.prot5408 20360362

[B20] Kim S. Y. Shin S. H. Moon S. M. Yang B. Kim H. Kwon O. J. (2017). Distribution and clinical significance of *Mycobacterium avium* complex species isolated from respiratory specimens. *Diagn. Microbiol. Infect. Dis.* 88 125–137. 10.1016/j.diagmicrobio.2017.02.017 28291631

[B21] Kozlov A. Diego D. Tomas F. Benoit M. Alexandros S. (2018). RAxML-NG: a fast, scalable, and user-friendly tool for maximum likelihood phylogenetic inference. *bioRxiv* [Preprint]. 10.1101/447110 PMC6821337 31070718

[B22] Langmead B. Salzberg S. L. (2012). Fast gapped-read alignment with Bowtie 2. *Nat. Methods* 9 357–359. 10.1038/nmeth.1923 22388286 PMC3322381

[B23] Lecorche E. Haenn S. Mougari F. Kumanski S. Veziris N. Benmansour H. (2018). Comparison of methods available for identification of *Mycobacterium chimaera*. *Clin. Microbiol. Infect.* 24 409–413. 10.1016/j.cmi.2017.07.031 28782649

[B24] Levesque S. Dufresne P. J. Soualhine H. Domingo M. C. Bekal S. Lefebvre B. (2015). A side by side comparison of bruker biotyper and VITEK MS: utility of MALDI-TOF MS technology for microorganism identification in a public health reference laboratory. *PLoS One* 10:e0144878. 10.1371/journal.pone.0144878 26658918 PMC4689555

[B25] Leyer C. Gregorowicz G. Mougari F. Raskine L. Cambau E. de Briel D. (2017). Comparison of Saramis 4.12 and IVD 3.0 Vitek MS matrix-assisted laser desorption ionization-time of flight mass spectrometry for identification of mycobacteria from solid and liquid culture media. *J. Clin. Microbiol.* 55 2045–2054. 10.1128/JCM.00006-17 28424252 PMC5483906

[B26] Li H. Handsaker B. Wysoker A. Fennell T. Ruan J. Homer N. (2009). Genome project data processing subgroup. 2009. The sequence alignment/map format and SAMtools. *Bioinformatics* 25 2078–2079. 10.1093/bioinformatics/btp352 19505943 PMC2723002

[B27] Murcia M. I. Tortoli E. Menendez M. C. Palenque E. Garcia M. J. (2006). *Mycobacterium colombiense* sp. nov., a novel member of the *Mycobacterium avium* complex and description of MAC-X as a new ITS genetic variant. *Int. J. Syst. Evol. Microbiol.* 56 2049–2054. 10.1099/ijs.0.64190-0 16957098

[B28] Neville S. A. Lecordier A. Ziochos H. Chater M. J. Gosbell I. B. Maley M. W. (2011). Utility of matrix-assisted laser desorption ionization-time of flight mass spectrometry following introduction for routine laboratory bacterial identification. *J. Clin. Microbiol.* 49 2980–2984. 10.1128/JCM.00431-11 21632894 PMC3147730

[B29] Page A. J. Taylor B. Delaney A. J. Soares J. Seemann T. Keane J. A. (2016). SNP-sites: rapid efficient extraction of SNPs from multi-FASTA alignments. *Microb. Genom.* 2:e000056. 10.1099/mgen.0.000056 28348851 PMC5320690

[B30] Pranada A. B. Witt E. Bienia M. Kostrzewa M. Timke M. (2017). Accurate differentiation of *Mycobacterium chimaera* from *Mycobacterium intracellulare* by MALDI-TOF MS analysis. *J. Med. Microbiol.* 66 670–677. 10.1099/jmm.0.000469 28504926

[B31] Rahi P. Prakash O. Shouche Y. S. (2016). Matrix-assisted laser desorption/ionization time-of-flight mass-spectrometry (MALDI-TOF MS) based microbial identifications: challenges and scopes for microbial ecologists. *Front. Microbiol.* 7:1359. 10.3389/fmicb.2016.01359 27625644 PMC5003876

[B32] Richter M. Rossello-Mora R. (2009). Shifting the genomic gold standard for the prokaryotic species definition. *Proc. Natl. Acad. Sci. U.S.A.* 106 19126–19131. 10.1073/pnas.0906412106 19855009 PMC2776425

[B33] Schweickert B. Goldenberg O. Richter E. Gobel U. B. Petrich A. Buchholz P. (2008). Occurrence and clinical relevance of *Mycobacterium chimaera* sp. nov., Germany. *Emerg. Infect. Dis.* 14 1443–1446. 10.3201/eid1409.071032 18760016 PMC2603105

[B34] Springer B. Stockman L. Teschner K. Roberts G. D. Bottger E. C. (1996). Two-laboratory collaborative study on identification of mycobacteria: molecular versus phenotypic methods. *J. Clin. Microbiol.* 34 296–303. 8789004 10.1128/jcm.34.2.296-303.1996 PMC228786

[B35] Tortoli E. Rindi L. Garcia M. J. Chiaradonna P. Dei R. Garzelli C. (2004). Proposal to elevate the genetic variant MAC-A, included in the *Mycobacterium avium* complex, to species rank as *Mycobacterium chimaera* sp. nov. *Int. J. Syst. Evol. Microbiol.* 54 1277–1285. 10.1099/ijs.0.02777-0 15280303

[B36] van Ingen J. Hoefsloot W. Buijtels P. C. Tortoli E. Supply P. Dekhuijzen P. N. (2012). Characterization of a novel variant of *Mycobacterium chimaera*. *J. Med. Microbiol.* 61 1234–1239. 10.1099/jmm.0.045070-0 22700551

[B37] van Ingen J. Kohl T. A. Kranzer K. Hasse B. Keller P. M. Katarzyna A. (2017). Global outbreak of severe *Mycobacterium chimaera* disease after cardiac surgery: a molecular epidemiological study. *Lancet Infect. Dis.* 17 1033–1041. 10.1016/S1473-3099(17)30324-9 28711585

[B38] Wick R. R. Judd L. M. Gorrie C. L. Holt K. E. (2017). Unicycler: resolving bacterial genome assemblies from short and long sequencing reads. *PLoS Comput. Biol.* 13:e1005595. 10.1371/journal.pcbi.1005595 28594827 PMC5481147

[B39] Yu G. Smith D. K. Zhu H. Guan Y. Lam T. T.-Y. (2017). ggtree: an R package for visualization and annotation of phylogenetic trees with their covariates and other associated data. *Methods Ecol. Evol.* 8 28–36. 10.1111/2041-210X.12628

